# An Inducible T7 Polymerase System for High-Level Protein Expression in Diverse Gram-Negative Bacteria

**DOI:** 10.1128/mra.01119-22

**Published:** 2023-01-16

**Authors:** Jennifer L. Greenwich, Melene A. Alakavuklar, Clay Fuqua

**Affiliations:** a Department of Biology, Indiana University, Bloomington, Indiana, USA; University of Maryland School of Medicine

## Abstract

A broad host range (BHR)-inducible T7 RNA polymerase system was developed, enabling induction with isopropyl-β-d-thiogalactopyranoside (IPTG), similar to the Escherichia coli strain BL21(DE3) protocol, but it is now applicable in a wide range of bacteria. This system allows for high protein yields and purification from diverse Gram-negative bacteria, including the native host.

## ANNOUNCEMENT

High-level expression of proteins for purification is commonly performed using Escherichia coli BL21(DE3). BL21(DE3) harbors a prophage (DE3) encoding the T7 RNA polymerase (gene *1*) that recognizes the *P*_T7_ promoter (TAATACGACTCACTATAG), which is under the control of an isopropyl-β-d-thiogalactopyranoside (IPTG)-inducible promoter (1). Purification of heterologous proteins from E. coli intrinsically removes them from their native physiological context and any endogenous modification or regulation. Ectopic expression of proteins in their native hosts is often used to study their native function, with expression under the control of a regulated promoter, commonly the *lac* promoter (*P_lac_*). While these systems drive expression, there are drawbacks to each. For example, purification of protein from the native host is often limited, with current systems providing insufficient expression. Here, we develop a bipartite broad host range (BHR) plasmid-based *P*_T7_ expression system. Similar to E. coli protein expression protocols, the protein of interest (POI) is expressed from the T7 promoter that is carried here on a pVS-based (spectinomycin resistance, Sp^r^) BHR plasmid (2). A second compatible plasmid (pBBR origin; gentamycin resistance, Gm^r^) provides the T7 polymerase gene, expressed from *P_lac_* and carrying *lacI^Q^*, allowing efficient regulation with IPTG (3, 4). An alternate T7 polymerase expression plasmid (IncP origin; tetracycline resistance, Tc^r^) was also created (5).

**FIG 1:**
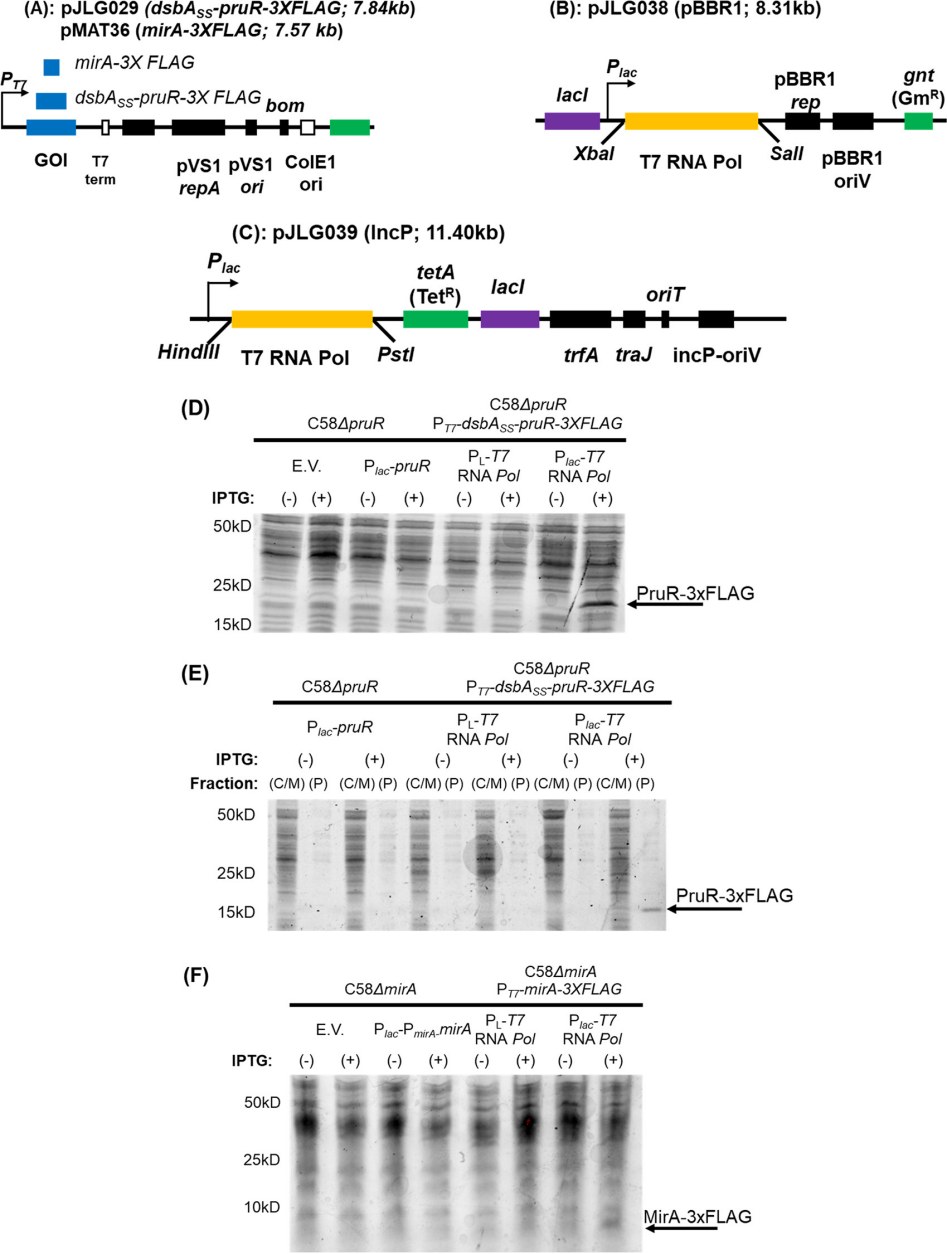
Plasmid maps and protein production in A. tumefaciens. Boxes with text indicate the relative position of genes. (A) Map of pJLG209 and pMAT36. The gene of interest is indicated in blue and the T7 terminator sequence in white. pVS1 *staA*, pVS1 *rep*, and pVS1 *ori* are a plasmid stability gene from the pVS1 plasmid, a replication protein, and the origin of replication, respectively (black). *Bom* is the basis of the mobility gene and is necessary for conjugation. The ColE1 ori is active in E. coli (white), and *aadA* encodes an aminoglycoside transferase (green), conferring resistance to spectinomycin and streptomycin. (B and C) Maps of pJLG038 and pJLG039. *lacI* (purple) encodes the lactose repressor, and T7 polymerase (gold) is ligated at the indicated sites. The origin, replication proteins, and conjugation proteins are indicated by black boxes. pBBR1 rep and pBBR1 ori encode the replication protein and origin, for pJLG038, *traJ* (for conjugation) and *incP-oriV*, and *oriT* for pJLG039. For antibiotic resistance genes (green), *gnt* encodes gentamicin acetyltransferase and *tetA* encodes a tetracycline efflux pump. (D) Expression of untagged PruR by the *lac* promoter or a DsbA_SS_-PruR-3XFLAG with IPTG-inducible *P*_T7_. (E) Periplasmic fractionation of the strains used in A (except for the negative control). (F) Expression of MirA, a cytoplasmic protein, using either *P_lac_* fused upstream of its native promoter (due to low yield from *P_lac_* alone) and a 3XFLAG tagged version of the protein under the control of the T7 promoter. E.V., empty vector; *P*_L_-T7 polymerase, constitutively expressed T7 polymerase; (C/M), cytoplasmic/membrane fraction; (P), periplasmic fraction.

The inducible T7 polymerase plasmid pJLG038 is a derivative of the BHR plasmid pSRKGm (derived from pBBR1MCS) (3, 4). To construct pJLG038, gene *1* was amplified from BL21(DE3) using forward (GTACTCTAGAATGAACACGATTAACATCGC [restriction enzyme sites are underlined]) and reverse (GTACGTCGACTTACGCGAACGCGAAGTCCG) primers and was ligated into pSRKGm digested with XbaI and SalI. The IncP plasmid was constructed in a similar manner. The plasmid was purified and sequenced before electroporation into Agrobacterium tumefaciens (6). The plasmids can also be introduced by conjugation (see Fig. 1A to C).

In addition to the inducible T7 polymerase plasmid, a second compatible plasmid carrying the POI is required. Here, we used derivatives of pRA301 (2) constructed using isothermal assembly with the POI fused to an efficient secretion signal (*dsbA*_SS_) and a 3XFLAG tag for purification.

While this work was performed and validated in Agrobacterium tumefaciens, the plasmids contain BHR replication origins and are compatible with a diversity of Gram-negative taxa (3, 4). With the aim to purify protein from A. tumefaciens, we discovered that available expression systems (based on *P_lac_*, *P*_N25_, and *P_traI_*) were insufficient for high-level protein expression (3, 7, 8). An existing system provided constitutive expression from the T7 polymerase in A. tumefaciens (9). However, we observed surprisingly weak expression and hypothesized that the constitutively expressed T7 polymerase negatively impacted cellular physiology, which has been noted in BL21(DE3) (10).

Unlike these systems, our IPTG-inducible T7 polymerase system provided tight regulation and high protein yields (Fig. 1D). No growth inhibition was observed during induction for the proteins tested. We initially designed and implemented this system to purify the A. tumefaciens periplasmic PruR protein (11), and periplasmic fractionation confirmed that the protein is efficiently targeted to the periplasm (Fig. 1E). The system is also effective for expression of cytoplasmic proteins, such as A. tumefaciens MirA (Fig. 1F) (12).

### Data availability.

The complete sequences of plasmids pMAT36 (P_T7_-*mirA*-3XFLAG, cytoplasmic protein), pJLG029 (P_T7_-*dsbA_SS_-pruR*-3XFLAG, periplasmic protein), pJLG038 (inducible T7 polymerase, pBBR, Gm^r^), and pJLG039 (inducible T7 polymerase, IncP, Tet^r^) have been deposited into GenBank (accession numbers OP627879, OP627880, OP627881, and OP627882) and are available from Addgene (identifier [ID] numbers 19279, 12980, 12981, and 12982). Plasmid inserts were sequenced using Sanger sequencing either through ACGT (Wheeling, IL) or Eurofins (Louisville, KY). These data are available upon request.

## References

[1] Studier FW, Moffatt BA. 1986. Use of bacteriophage T7 RNA polymerase to direct selective high-level expression of cloned genes. J Mol Biol 189:113–130. doi:10.1016/0022-2836(86)90385-2. 3537305

[2] Akakura R, Winans SC. 2002. Mutations in the occQ operator that decrease OccR-induced DNA bending do not cause constitutive promoter activity. J Biol Chem 277:15773–15780. doi:10.1074/jbc.M200109200. 11877409

[3] Khan SR, Gaines J, Roop RM, II, Farrand SK. 2008. Broad-host-range expression vectors with tightly regulated promoters and their use to examine the influence of TraR and TraM expression on Ti plasmid quorum sensing. Appl Environ Microbiol 74:5053–5062. doi:10.1128/AEM.01098-08. 18606801 PMC2519271

[4] Kovach ME, Elzer PH, Hill DS, Robertson GT, Farris MA, Roop RM, Peterson KM. 1995. Four new derivatives of the broad-host-range cloning vector pBBR1MCS, carrying different antibiotic-resistance cassettes. Gene 166:175–176. doi:10.1016/0378-1119(95)00584-1. 8529885

[5] Chen CY, Winans SC. 1991. Controlled expression of the transcriptional activator gene virG in Agrobacterium tumefaciens by using the Escherichia coli lac promoter. J Bacteriol 173:1139–1144. doi:10.1128/jb.173.3.1139-1144.1991. 1991713 PMC207234

[6] Morton ER, Fuqua C. 2012. Genetic manipulation of Agrobacterium. Curr Protoc Microbiol Chapter 3:Unit 3D.2. doi:10.1002/9780471729259.mc03d02s25. PMC3434950 22549163

[7] Wang Y, Mukhopadhyay A, Howitz VR, Binns AN, Lynn DG. 2000. Construction of an efficient expression system for Agrobacterium tumefaciens based on the coliphage T5 promoter. Gene 242:105–114. doi:10.1016/s0378-1119(99)00541-7. 10721702

[8] Danhorn T, Hentzer M, Givskov M, Parsek MR, Fuqua C. 2004. Phosphorus limitation enhances biofilm formation of the plant pathogen Agrobacterium tumefaciens through the PhoR-PhoB regulatory system. J Bacteriol 186:4492–4501. doi:10.1128/JB.186.14.4492-4501.2004. 15231781 PMC438617

[9] Zhu J, Chai Y, Zhong Z, Li S, Winans SC. 2003. Agrobacterium bioassay strain for ultrasensitive detection of N-acylhomoserine lactone-type quorum-sensing molecules: detection of autoinducers in Mesorhizobium huakuii. Appl Environ Microbiol 69:6949–6953. doi:10.1128/AEM.69.11.6949-6953.2003. 14602662 PMC262303

[10] Vethanayagam JG, Flower AM. 2005. Decreased gene expression from T7 promoters may be due to impaired production of active T7 RNA polymerase. Microb Cell Fact 4:3. doi:10.1186/1475-2859-4-3. 15638935 PMC545050

[11] Feirer N, Xu J, Allen KD, Koestler BJ, Bruger EL, Waters CM, White RH, Fuqua C. 2015. A pterin-dependent signaling pathway regulates a dual-function diguanylate cyclase-phosphodiesterase controlling surface attachment in Agrobacterium tumefaciens. mBio 6:e00156. doi:10.1128/mBio.00156-15. 26126849 PMC4488946

[12] Alakavuklar MA, Heckel BC, Stoner AM, Stembel JA, Fuqua C. 2021. Motility control through an anti-activation mechanism in Agrobacterium tumefaciens. Mol Microbiol 116:1281–1297. doi:10.1111/mmi.14823. 34581467 PMC8690355

